# Personal

**Published:** 1917-06

**Authors:** 


### PERSONAL

Dr. James Stoddart of Buffalo announces the removal of his office and residence to 160 W. Utica on May 1st.

At the Touraine Hotel April 23, the class of 1890 Niagara University gave a dinner in honor of Dr. J. Henry Dowd, a member of that class; Dr. John J. Finerty acted as toastmaster. Impromptu speeches were made by each of his classmates also by Mr. Edward G. Kirk, Supt. of the Pullman Car Co., who relieved Dr. Dowd in the office of Supt. Burrows of the N. Y. C. Ry. Co., he responded to the toast, J. Henry Dowd as a railroad official. A very neat little souvenir, containing a cartoon taken from the Buffalo Evening News, depicting him as a golf expert, a maker of mission furniture, also several verses describing him from graduation till the present day, was given the diners.

Dr. George W. Cottis of Jamestown sailed April 20 to work with the Harvard Medical Unit in France.

Dr. Stanley J. Brown of Mt. Morris was thrown out of his automobile and injured, May 7.

Dr. Burt J. Maycock and Dr. A. L. Benedict of Buffalo, were at the Chalfonte, Atlantic City in the latter part of April.

Dr. Thomas Bagley of Buffalo returned last month from a trip to N. Y., Washington and Miami.

Dr. Charles Haase of Elmira has equipped his office for X-ray work.

Dr. Arnold H. May of Buffalo has moved his residence to 625 W. Delavan Ave., continuing his offices at 37 Allen St.

Dr. John R. Honiss of 595 University Ave., Rochester, announces the limitation of his practice to the ear, nose and throat.

